# Development of a cultivation process for the enhancement of human interferon alpha 2b production in the oleaginous yeast, *Yarrowia lipolytica*

**DOI:** 10.1186/1475-2859-10-90

**Published:** 2011-11-02

**Authors:** Najla Gasmi, Atef Ayed, Billel Bel Hadj Ammar, Rim Zrigui, Jean-Marc Nicaud, Héla Kallel

**Affiliations:** 1 Unité de Biofermentation, Institut Pasteur Tunis, 13 place Pasteur, BP 74 1002, Tunis, Tunisie; 2 INRA, UMR1319 Micalis, Domaine de Vilvert, F-78352 Jouy-en-Josas, France; 3 CNRS, Micalis, Domaine de Vilvert, F-78352 Jouy-en-Josas, France

**Keywords:** *Yarrowia lipolytica*, POX2 promoter, induction strategy, high cell density culture, recombinant human interferon α2b

## Abstract

**Background:**

As an oleaginous yeast, *Yarrowia lipolytica *is able to assimilate hydrophobic substrates. This led to the isolation of several promoters of key enzymes of this catabolic pathway. Less is known about the behavior of *Y. lipolytica *in large bioreactors using these substrates. There is therefore a lack of established know-how concerning high cell density culture protocols of this yeast. Consequently, the establishment of suitable induction conditions is required, to maximize recombinant protein production under the control of these promoters.

**Results:**

Human interferon α2b (huIFN α2b) production in *Yarrowia lipolytica *was used as a model for the enhancement of recombinant protein production under the control of the oleic acid (OA)-inducible promoter POX2. Cell viability and heterologous protein production were enhanced by exponential glucose feeding, to generate biomass before OA induction. The optimal biomass level before induction was determined (73 g L^-1^), and glucose was added with oleic acid during the induction phase. Several oleic acid feeding strategies were assessed. Continuous feeding with OA at a ratio of 0.02 g OA per g dry cell weight increased huIFNα2b production by a factor of 1.88 (425 mg L^-1^) and decreased the induction time (by a factor of 2.6, 21 h). huIFN α2b degradation by an aspartic protease secreted by *Y. lipolytica *was prevented by adding pepstatin (10 μM), leading to produce a 19-fold more active huIFN α2b (26.2 × 10^7 ^IU mg^-1^).

**Conclusion:**

*Y. lipolytica*, a generally regarded as safe (GRAS) microorganism is one of the most promising non conventional yeasts for the production of biologically active therapeutic proteins under the control of hydrophobic substrate-inducible promoter.

## Background

Protein quality, functionality, quantity and space time yield are the most important factors to consider when choosing the expression system for heterologous protein production, especially for those of therapeutic interest.

In contrast to bacteria, yeasts have a great secretion potential including a strict quality control with the ability to perform complex post transcriptional modification [1,2]. In addition they are easier and less expensive to work with than insect or mammalian cells, and are easily adapted to fermentation processes. *Saccharomyces cerevisiae *is the earliest and most widely used host for the production of heterologous proteins; about 20% of the approved recombinant biopharmaceutical are produced using this yeast [1]. However, it was found to have some limitations such as low secretion capacities, low product yield, and hyperglycosylation.

A comparative study between *S. cerevisiae *and the alternative yeasts *K. lactis*, *S. pombe*, *H. polymorpha*, and *Y. lipolytica *on their capacity to secrete active forms of six fungal enzymes showed that all the examined alternative yeasts were more efficient than *S. cerevisiae*. The most attractive yeast, especially in terms of performance reproducibility, was *Y. lipolytica *[3].

This non conventional yeast has aroused a strong academic and industrial interest [4,5]. It is considered as non pathogenic and "generally regarded as safe" (GRAS) microorganism. *Y. lipolytica *was also distinguished for its ability to secrete naturally several metabolites and proteins in large amounts into the culture medium. But, probably the most important characteristic of *Y. lipolytica *as a host is the convenience of its secretory apparatus; high efficiency, co-translational pathway and low overglycosylation, in some regards closer to that of mammalian cells than to those of many yeasts [3,4,6]. Moreover, development of molecular and cellular tools and a wide range of host strains make *Y. lipolytica *one of the most attractive host for the expression of heterologous proteins [6].

Much is known about its behavior in large bioreactors, but most of the studies carried out have focused on the production of oil or citric acid by single cells [4,7]. Only a few reports have focused on the scale-up of recombinant protein production in this yeast [8,9]. On the other hand most of the reported proteins employed either *XPR2 *or hp4d promoter expression system. However, the use of these systems remains limited and may not be compatible with some heterologous proteins or with industrial culture conditions. The inducible *XPR2 *promoter is active only at pH above 6 and its full induction requires high levels of peptones in the culture medium whereas the second promoter is constitutive; this can be problematic when the product being expressed is toxic to the host [6,9]. To overcome these limitations efforts have been made to develop new alternative promoters. The promoter of acyl-CoA oxidases (POX2) was isolated by Juretzek et al. [10] in 2000; it was very strong and highly inducible by fatty acids such as oleic acid and repressed by glucose and glycerol. Despite these interesting features, the use of the POX2 promoter for the transcriptional control of foreign proteins was limited to shaken flasks scale and, as oleic acid serves the purpose of both inducer and source of carbon and energy, it is not straightforward to distinguish in these studies between the growth and protein production phases when trying to define the ideal conditions for induction [11,12]. There is therefore a lack of established know-how concerning the fermentation of this expression system.

In yeasts, the induction of gene expression by oleic acid was initially attributed to activation sequences, such as the oleate response elements found in the upstream regions of genes encoding enzymes involved in the lipid metabolic pathway [5,13]. One of the most important key parameters in this expression system is the oleic acid concentration. Monitoring and controlling this variable are important because high levels of this inductor substrate can be toxic to the cells and low levels of oleic acid may not be enough to initiate the POX2 transcription [14]. In order to maximize product yield and to achieve a high and reproducible product quality, the selection of the optimal oleic acid feeding strategy during the induction phase, and the production in a bioreactor under controlled conditions is necessary. Consequently, the establishment of suitable inducer concentration and the strategy used to conduct the culture are a key issue that must be taken into account when trying to maximize the production of heterologous proteins during fermentation [15,16].

The aim of this study is to establish an efficient strategy for heterologous proteins production at high cell density of *Y. lipolytica *under the control of the oleic acid inducible *POX2 *promoter. For this purpose huIFNα2b was used as a model.

huIFN α2b is one of the most studied types of interferon α, and is encoded by an intronless gene. It is a nonglycosylated 165-amino acid protein with two conserved disulfide bonds: Cys1-Cys98 and Cys29-Cys138 [17]. It has been shown to have antiproliferative and immunomodulatory effects on various cell types [18]. Recombinant huIFN α2b has been approved for use in the treatment of more than 14 diseases worldwide, including hairy-cell leukemia, condyloma acuminatum, Kaposi's sarcoma, hepatitis B and C [17,19].

In this study various feeding strategies were assessed to optimize recombinant huIFN α2b by *Y. lipolytica*. The influence of pre-induction biomass level on the production of the target protein was also investigated. This involved determining the effect of co-feeding strategies based on combinations of oleic acid and glucose and of different ways of adding oleic acid on POX2 induction and cell biomass viability. This study also aimed to find ways of preventing protein degradation and to demonstrate the effect of pepstatin addition on huIFNα2b degradation and biological activity.

## Results and Discussion

### Effect of glucose feeding during the induction phase

**Figure 1:**
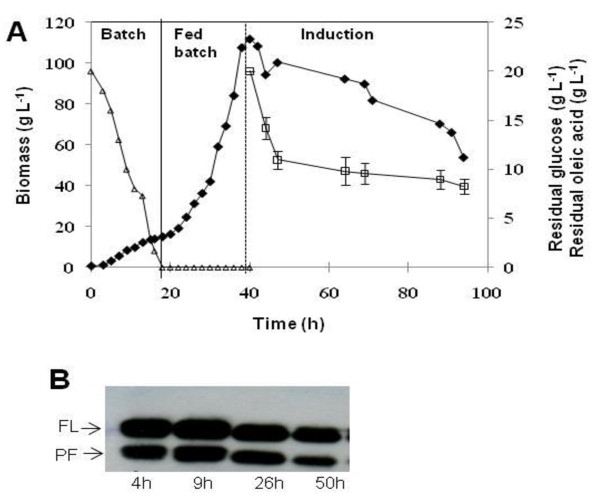
**Time course of biomass (closed diamond), glucose concentration (open triangle), and residual oleic acid concentration (open square) in a glucose exponential fed-batch culture**. The solid vertical line indicates the start of feeding, and the dotted vertical line indicates the addition of 20 g L^-1 ^oleic acid (A). Western blot analysis under reducing conditions with a polyclonal antibody against huIFN α2b (B). FL and PF correspond to the full-length and proteolytically degraded forms of huIFN α2b, respectively. Time (h) is the number of hours after induction with oleic acid. Bars indicate the mean values of two independent measurements.

A three-stage fed-batch culture technique was adopted for the production of huIFN α2b, using *Y. lipolytica *JMY1852p. Cells were first grown in batch mode in GNY medium with 20 g L^-1 ^glucose for 20 h, generating about 15 g L^-1 ^(DCW) biomass (Figure 1A).

After glucose depletion, a fed-batch phase with an exponential glucose feeding was started to generate high cell density concomitantly with derepression of the POX2 promoter. During the 20 h fed-batch culture, the specific growth rate of cells was maintained at 0.1 h^-1 ^(50% of μ_max_), a biomass concentration reached more than 110 g L^-1^. 20 g L^-1 ^of oleic acid was then added to initiate the induction phase. During this phase the biomass decreased slightly and then remained almost constant for the next 25 h (T = 65 h), after which it decreased strongly. Further extension of the induction phase led to cell lysis (Figure 1A).

Recombinant huIFN α2b production in the supernatant was checked at various time points by western blots analysis. Protein quantification revealed that huIFN α2b concentration peaked at 200 mg L^-1^. Protein production levels were highest during the first 9 h of induction, decreasing thereafter (Figure 1B). Two forms of huIFNα2b were produced: the full-length cytokine (FL) and a smaller, 14 kDa form probably resulting from proteolytic degradation (PF, Figure 1B).

A typical growth inhibition profile following the switch from glucose to oleic acid as the carbon source was observed during this culture (Figure 1A). Oleic acid does not seem to provide sufficient carbon and energy to sustain efficient growth and huIFNα2b production.

One approach to alleviate such problems is the use of a multicarbon substrate in addition to the inducer. This simple strategy increases the supply of energy to recombinant cells, decreasing the induction time and increasing volumetric productivity [15].

**Figure 2:**
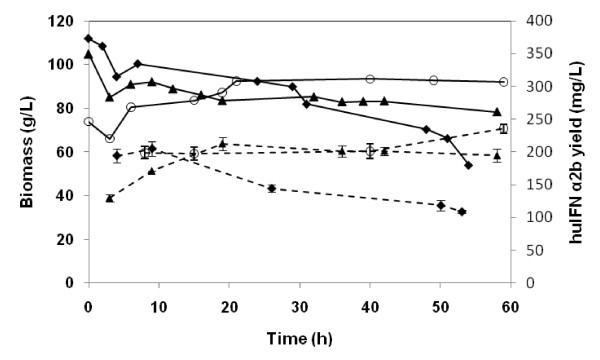
**Time course of biomass accumulation and huIFN α2b production during the induction phase**. Induction with oleic acid as the sole carbon source, and at an initial biomass level of 110 g L^-1 ^(closed diamond), induction using glucose as a cosubstrate with oleic acid and at a biomass level of 105 g L^-1^(closed triangle) and induction with glucose and oleic acid at an initial biomass level of 73 g L^-1 ^(open circle). Dotted lines indicate huIFN α2b production. Bars are the mean values of three different measurements.

Glucose was thus added at a constant flow rate of 4.8 g L^-1 ^during the induction phase. After an induction period of 58 h, final biomass decreased only to 80 g L^-1 ^following feeding with a combination of the two substrates, whereas it fell to 50 g L^-1 ^with OA alone (Figure 2). huIFNα2b concentration reached 212 mg L^-1 ^after 19 h of induction and contrary to the previous culture it remained almost constant for the rest of the culture period. Mean huIFNα2b specific productivity was 0.04 mg g^-1 ^DCW h^-1^.

The use of glucose as a co-substrate at a limited rate decreased cell lysis and promoted *Y. lipolytica* growth, as shown by the 33% increase in biomass and the 44% increase in huIFNα2b yield. These results are comparable to the enhancement of cell growth and the doubling of CD40 ligand production in *Pichia pastoris *reported by McGrew and coworkers [20] when mixed feeding with a 1:1 ratio of methanol to glycerol was used rather than methanol alone.

### Effect of pre-induction biomass level

The data reported above show that cell growth almost ceased in the induction phase, remaining very low until the end of the culture period, although the decrease in biomass was considerably reduced by the combined use of glucose and oleic acid during induction (Figure 2). This cell behavior may be due to space limitations when cell concentration rises beyond 105 g L^-1^.

Biomass at the start of the induction phase also played a crucial role in heterologous protein production. The effect of this parameter was investigated by setting up another culture in similar conditions but inducing when biomass reached 73 g L^-1^. Constant glucose feeding was applied during the induction phase. Biomass increased continually, reaching a maximum value of 94 g L^-1 ^at 40 h of induction (Figure 2). In addition, both the DCW yield on oleic acid and the amount of huIFN α2b were higher than those when the culture was induced at a biomass level of 105 g L^-1^. By contrast, mean huIFN α2b specific productivity was 39% higher than that for the culture induced at a biomass of 105 g L^-1^, possibly due to a better oxygen supply.

There have been conflicting reports concerning the effect of initial cell concentration before induction. Pinsach et al. [21] reported better *E. coli *growth and a higher maximum specific activity of rhamnulose-1-phosphate aldolase at lower ratios of IPTG to biomass. By contrast, Wang et al. [22] reported that low initial cell concentration at the start of induction resulted in a higher percentage of methanol assimilation into biomass, at the expense of polygalacturonate lyase production, in *Pichia pastoris*.

### Optimization of the oleic acid addition profile

A series of bioreactor cultures with similar growth phases on glucose was carried out to optimize the induction phase further, yielding high levels of huIFN α2b. Oleic acid was added at different initial concentrations (5, 10 and 20 g L^-1^) as a pulse or a continuous feed at a rate of 1.25 g h^-1^. For all cultures, biomass before induction was about 73 g L^-1 ^and constant feeding with glucose was maintained during the induction phase at 4.8 g h^-1^. Table 1 summarizes the induction strategies adopted.

**Table 1:** Comparison of kinetic parameters of huIFN α2b production under different induction strategies

Parameter	Strategy			
	
	1	2	3	4
S_t _(g L^-1^)	20	10	20	20
S_i _(g L^-1^)	20	10	5	^a^
Induction period (h)	55	54	33	21
S_c _(g L^-1^)	11.14	6.3	13.8	17.3
X (g L^-1^)	84.7	92.3	83.7	65
Y_X/S _(g g^-1^)	0.96	3.06	0.77	0
huIFNα2b (mg L^-1^)^b^	185	207	280	355
V (mg L^-1 ^h^-1^)	3.36	3.83	8.5	16.9
P (mg g^-1 ^h^-1^)	0.04	0.041	0.1	0.26

^a ^Oleic acid was add by a continuous feeding, at a rate of 1.25 g h^-1^

^b ^Mean level of huIFNα2b

S_t_: total oleic acid concentration, S_i_: initial oleic acid concentration, S_c_: substrate consumed. X: mean biomass level during induction, V: mean volumetric production of huIFN α2b, P: mean specific production of huIFN α2b

**Figure 3:**
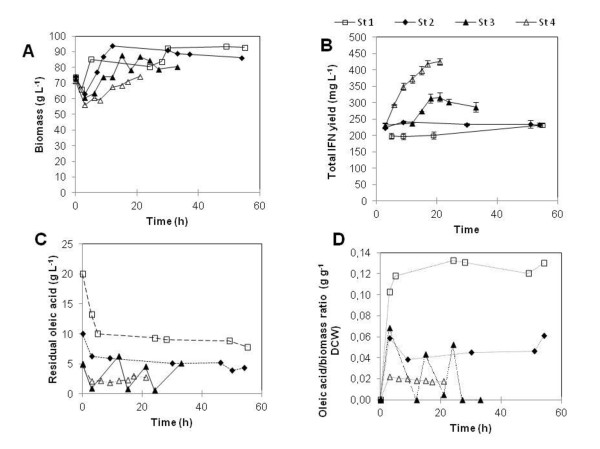
**Time course of biomass concentration (A), huIFN α2b production (B), residual oleic acid concentration (C) and oleic acid to biomass ratio (D) for the various strategies tested during the induction phase**. St 1, St 2, St 3 and St 4 correspond to a 20 g L^-1 ^oleic acid pulse, a 10 g L^-1 ^oleic acid pulse, 4 pulses of oleic acid, at 5 g L^-1 ^at 6 h intervals each, and continuous feeding with oleic acid at a rate of 1.25 g h^-1^.

The different induction strategies generated different growth features (Figure 3A): the values of (Y_x/s_) obtained were 0.96, 3.06, 0.77 and 0 for strategies 1, 2, 3 and 4, respectively.

huIFN α2b was first detected in the medium after a 3 h lag phase, for all cultures (Figure 3B). The production of this protein coincided with a slight decrease in biomass. This lag phase probably corresponds to the time required for the β oxidation of oleic acid. In Y. *lipolytica*, β oxidation enzymes are produced in the cytoplasm before being transported to the peroxisome, in which β oxidation occurs [23].

In the presence of 20 g L^-1 ^oleic acid (strategy 1: control culture), huIFNα2b concentration rapidly reached 200 mg L^-1^, gradually increasing thereafter to 240 mg L^-1 ^at the end of the induction phase. Oleic acid concentration fell rapidly to 9 g L^-1 ^at 9 h, remaining constant thereafter, corresponding to an oleic acid/biomass ratio of 0.12 (g g DCW^-1^). Following the addition of oleic acid to the medium at a concentration of 10 g L^-1 ^(strategy 2), the highest levels detected of huIFN α2b was 240 mg L^-1^. In these cultures, residual oleic acid concentration was 6-9 g L^-1 ^when the highest level of huIFN α2b was reached. Nutrient limitation did not cause the cessation of substrate uptake; in appropriate experiments, supplementation with complex medium did not improve oleic acid uptake (data not shown), probably due to the metabolic stress caused by the overburdening of the cell machinery at high oleic acid concentration.

Assuming that the amount of oleic acid transferred in strategies 1 and 2 was too high for an efficient protein production, the initial concentration of 20 g L^-1 ^was added as four pulses of 5 g L^-1 ^oleic acid at 6 h intervals, to avoid the accumulation of oleic acid in the medium (strategy 3). huIFN α2b content reached with this new strategy was considerably higher than that attained with the previous strategies. Production increased rapidly, reaching values of more than 315 mg L^-1 ^at 21 h of induction. Residual oleic acid concentration (Figure 3C) showed that the three first pulses were entirely consumed within three hours of the pulse. Nevertheless, the fourth pulse resulted in an accumulation of oleic acid and a slight decrease in the recombinant protein production level. No intracellular huIFN α2b was detected during this culture, suggesting that secretion of the protein was not a limiting factor (data not shown).

A culture in which oleic acid was continually added to the bioreactor, at a flow rate of 1.25 g h^-1 ^was then carried out. This approach increased huIFNα2b production to 425 mg L^-1 ^(Figure 3). This corresponds to twice the amount of huIFNα2b produced following pulses with oleic acid at 20 or 10 g L^-1^. Residual oleic acid remained below 2 g L^-1^.

The amount of oleic acid added during strategy 1 was clearly not optimal for the efficient production of huIFNα2b. However, when the same amount of oleic acid was fed continuously, it resulted in 84% higher levels of huIFNα2b specific production and a shorter induction time. The ratio of oleic acid per gram of biomass required to trigger huIFN α2b gene expression was only one sixth that required when oleic acid was in excess (strategy 1). These results suggest that the specific amount of oleic acid added (in grams of oleic acid per gram of DCW) and the way in which oleic acid is used are the most important variables to be considered for efficient induction. Furthermore, these results suggest that residual oleic acid concentration plays an important role in the process. These findings are consistent with those of Gordillo et al. [14], who described the use of oleic acid as an inducer for lipase production by *Candida rugosa*. They reported that the initial concentration of oleic acid had a major effect on final levels of lipolytic activity. The maximum lipase yield was obtained at an initial oleic acid concentration of 2 g L^-1^. At high concentrations, up to 8 g L^-1^, specific productivity decreased. They also showed that a minimal oleic acid concentration was required (1 g L^-1^) for lipase production.

Differences in protein production may be due to the kinetics of inducer uptake. The transport of oleic acid to the site of β oxidation requires morphological and physiological modifications. It was reported that an energy-free transporter was required for oleic acid uptake below a threshold of 10 μM, whereas for higher quantities oleic acid diffused freely. This might induce the POX2 promoter fully over a very short period of time [13]. Alternatively, these differences may be due to regulation of the enzymes of the β oxidation loop, potentially linked to substrate concentration. The presence of a long-chain fatty acid substrate in the medium stimulates POX2, and may repress POX3, which prefers short-chain fatty acids. The uptake of large amounts of oleic acid might therefore result in an accumulation of β-oxidation intermediates, leading to POX2 repression and an overload of subsequent β-oxidation steps [5].

### Analysis and control of huIFN α2b proteolysis

**Figure 4:**
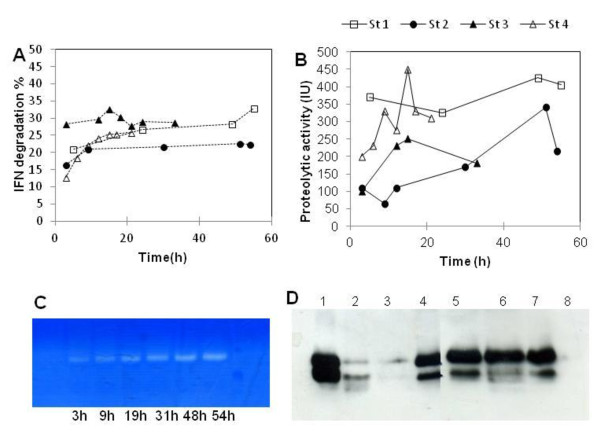
**Percentage huIFN α2b degradation (A) and protease activity (B) during the various induction strategies**. A 28 kDa protease detected in the supernatant for strategy 1 by zymography (C). huIFN α2b stability in the supernatant at various pH and in culture broth (D). Lanes 1-7 correspond to pH 2, pH 3, pH 4, pH 5, pH 6, pH 7, pH 8 and lane 8 corresponds to broth culture with protein synthesis inhibitors.

For all the cultures described above, two main bands were present on western blots: the upper band probably corresponding to the full-length protein and the lower band, at 14 kDa that could be a degraded product (Figure 1B). The ratio of the degraded form to the full-length form was estimated by scanning western blots (Figure 4A). The amount of the degraded form was proportional to production yield, accounting for 20 to 30% of total huIFN α2b despite the addition of casaminoacids (0.1 g L^-1^) every 24 h during the induction phase to reduce proteolysis. The nature of the proteases behind the cleavage of huIFN α2b was analyzed by zymography, and proteolytic activity was determined. No proteases were detected at the start of induction; these enzymes were first detected at 3 h and increased in amount during the production phase (Figure 4B and 4C). This cleavage was probably catalyzed by aspartic proteases, because it was completely inhibited by pepstatin (data not presented). The protease concerned is probably the acidic protease AXP, which is secreted by *Y. lipolytica *at low pH [6]. Furthermore, the *XPR2 *gene encoding the alkaline protease (AEP), which presents a potent threat to recombinant protein expression, was deleted from the Pold strain used in this work.

The detected protease had a high level of proteolytic activity (Figure 4B), the highest levels are coinciding to the peak huIFN α2b synthesis. AXP is less active at pH 5, the pH used in this study. Large amounts of this enzyme are produced to satisfy cellular requirements. This might account for the strong protease activities reported in all cultures.

huIFN α2b stability was studied by analyses on cell-free culture supernatant and culture broth withdrawn from the bioreactor and incubated in the presence of protein synthesis inhibitors, as described in the materials and methods section. huIFN α2b yield was significantly affected at pH 3 and 4 (Figure 4D). However, protein yields were also slightly affected at pH values above 5, during the three days of incubation at 28°C. These data confirm the sensitivity of huIFN α2b to AXP and are consistent with the optimal pH for AXP (around 3) and with data reported by Glover et al. [24] who showed that this protease was less active at pH 5 to 6.

huIFN α2b losses were much greater after the addition of protein synthesis inhibitors to culture broth; this suggests that both secreted and cell-associated proteases contributed to huIFN α2b degradation. The reaction was faster when cells were present (Figure 4D, line 8). This may explain the culture profile observed in Figure 2, which shows a marked decrease in the amount of huIFN α2b during cell lysis.

**Figure 5:**
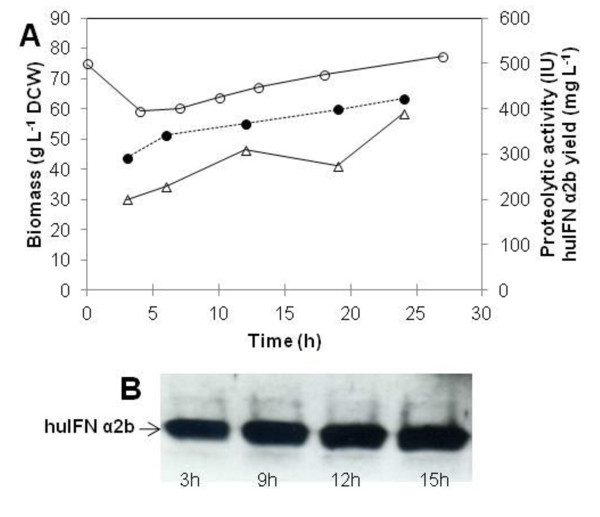
**High-density culture of *Y. lipolytica *producing huIFN α2b**. During induction, oleic acid was added continuously to the bioreactor at a flow rate of 1.25 g h^-1^; pepstatin was added, at a concentration of 10 μM, at the start of induction. Time course of biomass (open circle), huIFN α2b production (closed circle) and protease activity (open triangle) (A). Western blot analysis showing huIFN α2b as a single full-length form (B).

The problem of this proteolysis was overcome by performing another culture, to which pepstatin was added at a single dose of 10 μM at the beginning of the induction phase. The influence of pepstatin on growth, huIFN α2b production and proteolytic activity is shown in Figure 5A. The addition of pepstatin to the culture medium had no negative effect on growth rate, but increased biomass slightly. Moreover, the target protein was produced in a single form, corresponding to the full-length huIFN α2b (Figure 5B), suggesting that the proteolytic activity of the AXP protease was totally inhibited and confirming that the lower band was not a truncated polypeptide resulting from low levels of transcription. However, no further increase in the amount of huIFN α2b was obtained, with the maximum yield obtained from this culture similar to that obtained without pepstatin. One way to avoid protein degradation is to use Po1f strain of *Y. lipolytica *that is lacking both AEP (alkaline extracellular protease) and AXP [6].

### huIFN α2b biological activity

**Figure 6:**
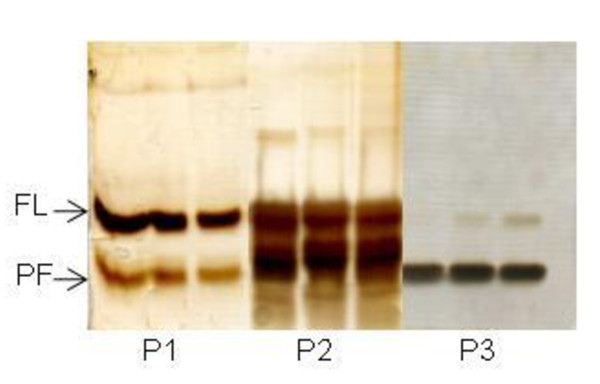
**Silver-stained SDS-PAGE of samples collected during the size exclusion purification step**. P1, P2 and P3 represent the pooled fractions tested for biological activity. FL: full-length form and PF: proteolytically degraded form.

The two forms of huIFN α2b were eluted, at the same ionic strength, from cation exchange and gel filtration columns, and had different estimated molecular weights on silver nitrate-stained SDS PAGE gels (Figure 6).

N-Terminal sequencing was performed on both products. The sequence for the higher molecular weight band (C-D-L-P-Q-T) corresponded to the N-terminal sequence of huIFN α2b. The lower molecular weight band had the following N-terminal sequence: I-S-L-F-S-C, corresponding to the huIFNα2b protein lacking 23 amino acids. The biological activity of the purified huIFN α2b was assessed and compared with that of the huIFN α2b produced in *Pichia pastoris*. The huIFN α2b produced by *Y. lipolytica *was biologically active. The level of biological activity was found to depend on the level of proteolytic degradation of the produced huIFN α2b (Table 2). When full-length huIFN α2b predominated, the IFN produced by *Y. lipolytica *was about twice (26.2 × 10^7 ^IU mg^-1^) as that produced by *Pichia pastoris*.

**Table 2:** Biological activity of huIFNα2b purified from *Y.lipolytica*

Pool	Composition	Biological activity (IU mg^-1^)
P1	Full-length: +++	26.2 × 10^7^
	Cleaved form: +	
P2	Mixture	7.47 × 10^7^
P3	Full-length: +/-	1.41 × 10^7^
	Cleaved form: +++	

+++: predominant; +: present; +/-: minor

It has been reported that only the second disulfide bond is essential for the biological activity of huIFN. X-ray crystallography showed that the huIFN α2b monomer consisted of five helices, A to E, linked by one overhand connection (AB loop) and three short segments. Two regions of huIFN α2b were identified as critical for biological activity: the high-affinity receptor binding site 1, consisting of residues on helix A, the AB loop and helix E, and a second site formed from various residues on helix A and helix C [25]. However, N-terminal sequencing showed that helix A was degraded in the cleaved form of huIFN α2b generated by *Y. lipolytica*. This may account for the data presented in Table 2.

## Conclusion

In conclusion, huIFN α2b production under control of the POX2 promoter was significantly enhanced by an optimized cofeeding and induction strategy. The lower levels of cell lysis and higher levels of huIFNα2b when glucose and oleic acid were supplied together suggest a positive effect on POX2 expression, oleic acid transport and huIFNα2b secretion. It may be possible to optimize this cofeeding method further and to apply it for the production of other heterologous proteins. This study demonstrates that *Y. lipolytica *is an efficient host for the production of biologically active therapeutic proteins.

## Materials and methods

### Strain

The *Y. lipolytica *strain JMY1852p is a prototrophic derivative of JMY1852 [26], which harbors the JME1070 vector for expression of the codon-optimized huIFN α2b gene under the control of the strong, OA-inducible POX2 promoter. huIFNα2b secretion is directed by the targeting sequence of the *Y. lipolytica *extracellular lipase (Lip2p) pre-signal peptide.

### Media

The recombinant strain was isolated on YPD-agar (yeast peptone dextrose; 20 g L^-1 ^glucose, 10 g L^-1 ^yeast extract, 10 g L^-1 ^peptone and 20 g L^-1 ^agar) plates. The GNY minimal medium used in this study has been described elsewhere [27]. Briefly this medium is composed of basal salt solution (CaSO_4 _2H_2_O, 0.93 g L^-1^; K_2_SO_4_, 18.2 g L^-1^; MgSO_4 _7H_2_O, 7.28 g L^-1^; KOH, 4.4 g L^-1^; glucose, 20 g L^-1 ^and H_3_PO_4 _85%, 26.7 mL L^-1^) supplemented with 10 mg L^-1 ^FeCl3, 1 g L^-1 ^glutamate, 5 ml L^-1^, 5 ml L^-1 ^of PTM1 oligo-elements solution (CuSO_4 _5 H_2_O, 6 g L^-1^; KI, 0.08 g L^-1^; MnSO_4 _2H_2_O, 3 g L^- 1^; Na_2_MoO_4 _2H_2_O, 0.2 g L^-1^; H_3_BO_3_, 0.02 g L^-1^; CoCl_2 _6H_2_O, 0.5 g L ^-1^; ZnCl_2_, 20 g L^-1^; FeSO_4 _7H_2_O, 6.5 g L^-1^. H_2_SO_4_, 5 mL L^-1^) and 2 ml L^-1 ^vitamin solutions (Biotin, 8 μg L^-1^; Thiamin, 200 μg L^-1 ^and myo-inositol 4 μg L^-1^). The fed-batch solution contained 666 g L^-1 ^glucose and PTM1 (5 ml L^-1^). Vitamins (2 ml L^-1^) were added to this solution after heat sterilization.

The stock solution (20% oleic acid, 0.5% Tween 20) was subjected to sonication for 2 min with a BANDELIN SONOPLUS sonicator (Berlin, Germany) for emulsification. Vitamins (2 ml L^-1^) were added to the fed-batch oleic acid solution.

### Bioreactor cultures

The preinoculum culture was grown from a single colony isolated on YPD-agar plates, in a 250 mL flask containing 20 mL YPD medium and incubated overnight at 28°C, with shaking at 180 rpm, in a rotary shaker. The preinoculum was transferred to a 2 L baffled flask containing 200 mL of GNY medium and incubated at 28°C for 12 h, with shaking at 180 rpm. For batch bioreactor culture, the 200 mL of inoculum culture was transferred aseptically to the bioreactor, which contained 2 L of GNY medium. All cultures were carried out in a 5 L stirred bioreactor (Infors, Bottmingen, Switzerland). The pH was kept at 5 by adding 25% ammonia solution to the reactor, and the temperature was maintained at 28°C. Dissolved oxygen levels were kept at 40% of air saturation, by adjusting the stirrer speed between 600 and 1000 rpm and supplying air and/or pure oxygen. Bioreactor cultures were initiated by a batch growth phase on glucose for approximately 20 h. The kinetic parameters of cell growth were determined during this phase. The coefficient yield of glucose conversion to biomass (Y_X/S_) was 0.75 g g^-1 ^(DCW on glucose), the maximum specific growth rate (μ_max_) was 0.22 h^-1 ^and the maintenance term (m) was 0.06 g g^-1 ^h^-1^.

The end of the batch phase was identified by a decrease in oxygen consumption rate. A simple mathematical model based on mass balances and substrate consumption kinetics was used to keep the specific growth rate constant, to ensure exponential growth on glucose [28]. Induction was performed by adding oleic acid solution in various ways; unique pulses of 10 or 20 g/L, repetitive pulse of 5 g/L, and 1.25 g/L continuous feeding depending on the experiment. Casaminoacids (Bacto, France) were added to the fermentor at a concentration of 0.1 g L^-1 ^every 24 h during the induction phase.

### SDS-PAGE and western blot analysis

Sodium dodecyl sulfate polyacrylamide gel electrophoresis (SDS-PAGE) was performed in 15% polyacrylamide gels under denaturating conditions, as described by Laemmli [29]. After separation, proteins were stained with either Coomassie brilliant blue R 250 or silver nitrate.

For western blot analysis, 10 μl of diluted supernatant were separated by SDS-PAGE and transferred onto nitrocellulose membranes (Millipore, Bedford, MA, USA) by electroblotting. The membranes were blocked with PBS-5% skimmed milk, 0.1% Tween 20 overnight at 4°C. Membranes were incubated for 1 hour with anti-human IFNα2b polyclonal antibody produced in-house and diluted to 1/200 followed by incubation with a goat antimouse IgG peroxidase conjugated monoclonal antibody (Sigma, St Louis, USA) diluted at 1/5000. The immunoreactive protein was visualized by ECL (GE Healthcare, Uppsala, Sweden).

### Analytical methods

Cell concentration was estimated from the optical density at 600 nm of an appropriately diluted culture sample. The correlation between OD_600 _and dry cell weight was determined according to standard protocols. After growth on medium containing oleic acid, samples were extracted with a 2/5 (*v/v*) mixture of propanol/butanol before optical density determination. One OD_600 _unit was equivalent to 0.35 g L^-1 ^dry cell weight (DCW).

Glucose concentration was determined in an enzymatic assay (kit from Eurodiag, France). Oleic acid concentration was estimated by the colorimetric method, based on a sulfo-phospho-vanillin reaction described by Frings and Dum [30].

Protease activity was determined either by the colometric method using azocasein as a substrate or by zymography analysis. Culture supernatant (10 μl) was mixed with 10 μl of a 2.5% azocasein solution and 70 μl of 0.1 M phosphate-citrate buffer pH 5 then incubated at 28°C for 1 h. The reaction was stopped by addition of 350 μl of 10% TCA (Trichloro acetic acid) solution. Samples were centrifuged at 13.000 rpm for 10 min, and then the absorbances of the supernatant were read at 440 nm against the blank. One unit of protease activity was defined as the amount of enzyme required for an increase in absorbance by 0.01 per hour. Zymography analysis was carried out as previously described [27]. Briefly, 15% separating gels were mixed with 5 mg casein; samples were treated with three-fold concentrated sample buffer. Gels were run at constant current of 100 mV. Afterwards the gels were rinsed three times with 2.5% (V/V) Triton X-100 and incubated overnight in 50 mM acetate buffer pH 5 with or without inhibitors. Gels were stained with Coomassie blue and then destained until transparent zones caused by proteolytic digestion of the protein substrate in the gel, are visible against a blue background.

huIFN α2b production was determined by scanning and using J-image software (Image-J 1.42) to analyze the area of each band on western blot membranes. Concentrations were then calculated from a calibration curve established with purified huIFN-α2b produced in *Pichia pastoris*. The results shown are the mean values of three scans.

The N-terminal amino acid sequences of the two of huIFN-α2b forms were determined with the 420 A-130 A Derivatizer-Analyzer System (Applied Biosystems) after hydrolysis in 6 M HCl at 110°C for 24 h. The N-terminus of each form was sequenced by automated Edman degradation, with an Applied Biosystems 492 Procise protein sequencer equipped with a PTH 140C analyzer.

### Proteolysis kinetics

For the analysis of huIFN α2b degradation in the culture broth, cycloheximide (20 mg L^-1^) and chloramphenicol (200 mg L^-1^) were added to 25 mL of the culture broth taken from the bioreactor 9 h after induction, to prevent *de novo *synthesis of proteins. Samples were incubated in flasks at culture temperature (28°C), shaken at 180 rpm. Samples were regularly withdrawn to determine the level of huIFN α2b degradation.

The influence of pH on huIFN α2b stability was investigated by analyzing culture supernatants collected during bioreactor culture 20 h after induction. The pH was adjusted to 2, 3, 4, 5, 6, 7 and 8, by adding 85% H_3_PO_4 _or 25% NH_4_OH before incubation at 28°C and 180 rpm. Samples were withdrawn at various time points and used for western blotting.

### Purification of huIFN α2b

Culture broth collected at the end of the bioreactor culture was centrifuged, clarified by 8 μm filtration and desalted on a Sephadex G-25 column. huIFN α2b was then purified on a cation exchange column, followed by a Sephacryl S-100 column, as described elsewhere [31].

### Biological activity

The biological activity of the *Y. lipolytica *huIFNα2b preparation was determined with the SEAP reporter gene assay, as described by Meager [32]. HEK (human embryo kidney) 293P cells transfected with a reporter plasmid encoding the secreted embryonic alkaline phosphatase (SEAP) under the control of an IFN-inducible promoter sequence (ISRE, interferon-stimulated response element) were grown in 96-well plates, at a density of 10^5 ^cells mL^-1 ^for 24 h and were stimulated with various dilutions of huIFNα2b. After incubation for a further 48 h, supernatants were collected and assayed for SEAP activity, using para nitrophenyl phosphate as the substrate. huIFN α2b reference (code: 95/566) was used as a standard.

## Declaration of competing interests

The authors declare that they have no competing interests.

## Authors' contributions

NG carried out the experiments and drafted the manuscript. AA participated in bioreactor cultures and biological activity tests. BHA and RZ participated in bioreactor cultures. JMN supervised clones design and reviewed the manuscript. HK conceived the study and reviewed the final manuscript. All authors read and approved the final manuscript.
